# Optimal Selection of the Effective Echo Time (TE_eff_ ) for T2‐Weighted Fast‐Spin‐Echo MRI of the Prostate at 3.0 T: Effect on Lesion Conspicuity

**DOI:** 10.1002/jmri.70351

**Published:** 2026-04-23

**Authors:** Dahan Kim, Ronard Pabi, Eric A. Borisch, Adam T. Froemming, Roger C. Grimm, Sara Hassanzadeh, Akira Kawashima, Ayumu Kido, Naoki Takahashi, John V. Thomas, Stephen J. Riederer

**Affiliations:** ^1^ Department of Radiology Mayo Clinic Rochester Minnesota USA; ^2^ Department of Physics Gustavus Adolphus College St. Peter Minnesota USA; ^3^ Department of Radiology Mayo Clinic Phoenix Arizona USA

**Keywords:** echo time, prostate, *T*_2_‐weighted

## Abstract

**Background:**

Clinical *T*_2_‐weighted (T2W) MRI of prostate cancer (PCa) usually implements a 2D fast‐spin‐echo (FSE) sequence, but understanding the echo‐time‐dependent contrast behavior is not straightforward due to the complicated FSE signal evolution. Consequently, consensus‐based recommendations on the optimal effective echo time (TE_eff_) to maximize contrast are lacking.

**Purpose:**

To investigate the effect of TE_eff_ on PCa lesion contrast in prostate T2W MRI.

**Study Type:**

Phantom and in vivo prospective study.

**Subjects:**

A standard MRI phantom and 53 prostate cancer patients (29 for image evaluation and 24 for quantitative lesion analysis).

**Field Strength and Sequence:**

Multi‐slice T2W FSE sequence at 3 T acquired at multiple TE_eff_ values.

**Assessment:**

Three readers (13, 25, and 27 years' experience) evaluated TE_eff_ = 100 ms versus 150 ms on diagnostic quality and reader preference. The effective *T*_2_ relaxation rates (*T*_2,eff_) were measured using FSE sequences in the phantom and used to predict the optimal TE_eff_ maximizing T2W contrasts. From multi‐TE_eff_ scans of subjects with suspected PCa, *T*_2,eff_ values of PCa lesions and healthy tissue were measured and used to calculate the optimal TE_eff_ maximizing the lesion contrast. In vivo contrast metrics were measured as a function of TE_eff_.

**Statistical Tests:**

Wilcoxon test of reader scores, with *p* < 0.05 defining statistical significance.

**Results:**

No statistically significant difference was found in diagnostic quality between TE_eff_ = 150 ms and 100 ms images, but all three readers significantly favored TE_eff_ = 150 ms. Phantom experiments demonstrated that TE_eff_‐dependent FSE signal progression can be characterized by *T*_2,eff_ values typically 40% longer than actual *T*_2_ values, allowing accurate prediction of optimal TE_eff_ within 2%–5%. Applied to PCa imaging, in vivo measurements indicated that effective *T*_2_ relaxation times of the prostate are on average 1.5× longer than published *T*_2_ values.

**Data Conclusion:**

The benefits of TE_eff_ = 150 ms versus 100 ms in prostate T2W FSE were supported by reader preference, phantom experiments, in vivo measurements, and analytic optimizations.

**Evidence Level:**

2.

**Technical Efficacy:**

2.

## Plain Language Summary

In *T*_2_‐weighted MRI of prostate cancer, the optimal value for the effective echo time maximizing lesion contrast is not well understood, as the sequence has complicated signal evolutions. Through phantom experiments and in vivo measurements of prostate cancer lesions, this study found that signal in the *T*_2_‐weighted prostate MRI decays typically 40% more slowly than the standard decay rates, extending the optimal effective echo time beyond the employed range reported in the literature. Accordingly, the reader preference and in vivo lesion contrast analysis of this study demonstrated the benefits of using higher effective echo times in *T*_2_‐weighted prostate MRI.

## 1 Introduction

**FIGURE 1:**
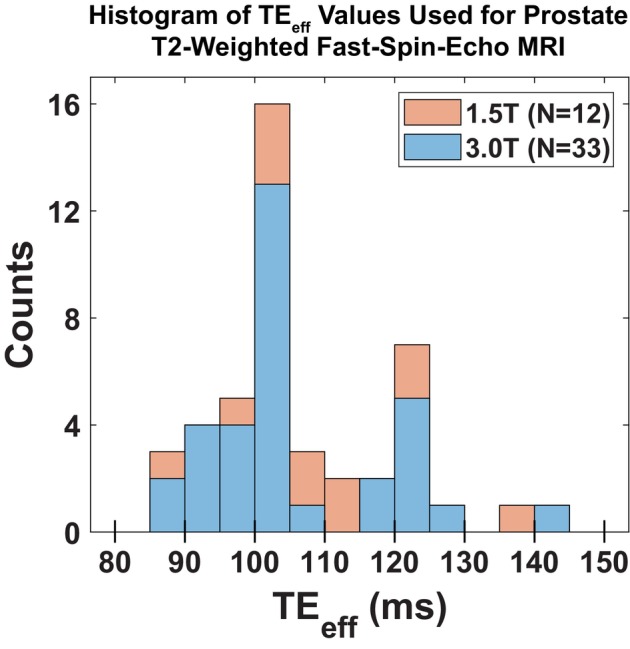
Histogram of TE_eff_ values reported in studies published between 2004 and 2024 for *T*_2_‐weighted fast‐spin‐echo imaging of the prostate. Thirty‐three of the 45 studies were performed at 3 T and 12 were at 1.5 T. A full listing of the references is provided in Table S1.

*T*_2_‐weighted (T2W) imaging plays a central role in MRI of prostate cancer (PCa) as a sequence to provide high‐contrast, high‐resolution morphological images. The T2W contrast of these images helps define lesion boundaries and prostate zonal anatomy, aiding accurate lesion localization and staging of PCa. The Prostate Imaging—Reporting and Data System (PI‐RADS) [1, 2], currently version 2.1, provides guidelines for the acquisition and interpretation of the component sequences of multiparametric MRI (mpMRI). For T2W, this includes, for example, specifications for in‐plane spatial resolution, slice thickness, and field‐of‐view, while also indicating that T2W images should be acquired in the axial and at least one other orientation. PI‐RADS further indicates that the T2W acquisition is generally performed with a fast‐spin‐echo (FSE) or turbo‐spin‐echo (TSE) sequence in which data are collected at multiple echo times along an echo train. The degrees of *T*_2_ weighting and lesion contrast in these T2W acquisitions are characterized by an effective echo time, TE_eff_. However, PI‐RADS is silent on TE_eff_ selection, and the optimal TE_eff_ value to maximize contrast in PCa imaging is unclear. Consequently, and perhaps not surprisingly, prostate MRI studies report a broad range of TE_eff_ values for the T2W sequence. For example, values ranging from 85 ms to 150 ms (mean/median = 106/104 ms) were found in a survey of 45 representative prostate MRI studies published between 2004 and 2024 from various MRI‐related journals (Figure 1). These studies are tabulated in Table S1.

Few previously published studies have discussed the selection of TE_eff_. In one previous study [3] at 3 T, three readers independently identified suspected PCa lesions in multi‐TE (TE = 90, 150, and 180 ms) prostate T2W images of 38 patients and compared MRI findings against histology results after radical prostatectomy. Two of the three readers attained significant improvement in sensitivity for PCa detection at TE = 180 versus 90 ms. Hence, this study suggested that the use of high TE values may be beneficial. An additional study examined PCa lesion contrast as a function of TE using a 3D GRASE sequence [4].

The lack of study of an optimal TE_eff_ for prostate T2W FSE imaging may stem from the fact that FSE has complicated echo pathways that do not lend themselves to analytic equations for TE_eff_‐dependent signal or contrast. However, as demonstrated below, the FSE sequence using a constant refocusing flip angle produces a signal decay that can be closely approximated by an exponential decay at an “effective” *T*_2_ relaxation time (*T*_2,eff_). As most prostate T2W exams utilize a constant‐flip‐angle FSE sequence, in vivo *T*_2,eff_ measurements of different prostate components could potentially allow calculation of the optimal TE_eff_ at which the lesion contrast maximizes, analogous to TE optimization for a conventional spin‐echo (SE) sequence using known *T*_2_ values.

Thus, the aim of this study was to determine the dependence of contrast on TE_eff_ in T2W prostate MRI and help characterize an optimal TE_eff_ value for maximum PCa lesion contrast.

## 2 Materials and Methods

### 2.1 MRI Equipment

All imaging studies, human and phantom, were performed at 3 T (Discovery MR750w and Premier, GE Healthcare, Waukesha, WI, USA).

### 2.2 Initial Comparison of Prostate T2W Imaging at TE_eff_ = 150 ms Versus 100 ms

This investigation into optimization of TE_eff_ in prostate T2W was triggered by an initial study in which a newly developed modified (MOD) T2W sequence was evaluated to possibly replace our institution's routinely used PI‐RADS v2.1‐adherent (ADH) T2W sequence. Sequence parameters are shown in Table 1. The MOD sequence has less anisotropic in‐plane spatial resolution, allowing a typical 40 s reduced acquisition time and is being evaluated for potential reduced motion sensitivity. At the time of comparison of the two sequences, we failed to note the different TE_eff_ times, 100 ms for MOD and 150 ms for ADH.

**TABLE 1:** Acquisition parameters of PI‐RADS‐Adherent (ADH) and Modified (MOD) *T*_2_‐Weighted Fast‐Spin‐Echo (FSE) sequences, used for human subject studies (initial reader evaluation and lesion analysis).

Parameter	ADH	MOD
Freq. × phase directions^a^	A/*P* × L/R	A/*P* × L/R
Field‐of‐view (mm)	160 × 160	160 × 160
Slice thickness (mm)	3	3
Slice‐to‐slice increment (mm)	3 (contiguous)	3 (contiguous)
In‐plane sampling	400 × 230	320 × 280
In‐plane resolution (mm)	0.40 × 0.70	0.50 × 0.57
In‐plane pixel area (mm^2^)	0.280	0.285
Reconstructed in‐plane pixel size (mm^2^)	0.156 × 0.156	0.156 × 0.156
Signal bandwidth (kHz)	±64.0	±32.0
Echo spacing (ESP) (ms)^b^	8.2	10.2
Echo train length (ETL)	24	21
Sampling time per slice per repetition (ms)	217	230
Repetition time (TR) (mm) (15 slices per pass)	3255	3450
Refocusing flip angle (°)	130	130
Effective echo time (TE_eff_) (ms) (initial reader evaluation)^b^	150	100
Effective echo time (TE_eff_) (ms) (lesion analysis study)^b^	—	100, 150, and additional values
Acceleration factor (ARC)	1.5	1.5
Number of averages	2	1
No phase wrap (phase encode oversampling)	2.00	2.00
Scan time per pass of 15 slices	46 s (14 repetitions)	69 s (20 repetitions)
Total scan time for 30 slices with averaging	3:02	2:18

^a^ A/P: anterior/posterior; L/R: left/right.

^b^ Shown for the prescribed values. Echo spacing and thus the actual TEeff and total scan time can change ±5 ms with oblique angulation.

To assess the potential for the MOD sequence to replace the ADH sequence for clinical prostate MRI, the MOD sequence was added to the routine protocol for clinical prostate MRI and performed immediately after the ADH sequence when allowed by the clinical schedule. Any study in which the subject was post‐prostatectomy was excluded from further consideration. All 29 resultant studies performed from April 5 to 12, 2024 were then evaluated by three radiologists (A.Ka., N.T., A.T.F.), with respective 25, 27, and 13 years' experience in prostate MRI. Demographics of the 29 participants are shown in Table 2. Results were evaluated to determine if the MOD sequence continued to provide diagnostic quality (DQ) similar to that of the ADH sequence as well as to assess any reader preference.

**TABLE 2:** Demographics of subjects for both human subject groups studied.

	TE_eff_ = 100 versus TE_eff_ = 150; *n* = 29		Lesion analysis; *n* = 24	
Dates of accrual	April 5 to 12, 2024		July 11, 2024 to August 11, 2025	
Age (years)	Median: 69, range: 46–88		Median: 65.5, range: 57–79	
Weight (kg)	Median: 94.3, range: 64.8–120		Median: 85.3, range: 60–117	
BMI (kg/m^2^)	Median: 28.2, range: 20.1–36.3		Median: 26.95, range: 20–34.6	
PSA (ng/ml)	Median: 5, range: 0.15–32.7		Median: 6.25, range: 1.5–12.8	
Prostate volume (cc)	Median: 44.5, range: 13.1–178		Median: 46, range: 25–125	
Diagnosis/Treatment	Treatment‐naïve; *n* = 24	PI‐RADS 2, *n* = 11	Treatment‐naïve; *n* = 24	PI‐RADS 2, *n* = 2
		PI‐RADS 3, *n* = 5		
		PI‐RADS 4, *n* = 4		
				PI‐RADS 3, *n* = 7
		PI‐RADS 5, *n* = 4		
	Post‐treatment for prostate cancer; *n* = 4	Cryo‐ablation, *n* = 2		
		Post radiation therapy + Cryoablation, *n* = 1		PI‐RADS 4, *n* = 13
		Chemotherapy + radiation therapy+ ADT+ cryo‐ablation, *n* = 1		
				PI‐RADS 5, *n* = 2
	Other; *n* = 1	Post transurethral resection of bladder tumor, *n* = 1		

Abbreviations: BMI, Body mass index; PSA, Prostate specific antigen.

Diagnostic Quality was assessed using a four‐point (0‐to‐3) scale developed and used by our reviewers in multiple previous evaluations [5, 6, 7, 8] and defined as follows:

- DQ 0 = obviously non‐diagnostic, severely limited by markedly poor SNR, gross motion or other artifact, or otherwise non‐interpretable;
- DQ 1 = marginally non‐diagnostic, with SNR, resolution, or artifact limiting interpretation, generally requiring a rescan;
- DQ 2 = non‐ideal in quality such as due to slight blurring but still allowing interpretation;
- DQ 3 = high diagnostic quality.

Prior to the evaluation, for calibration all three readers were presented with four exams at each DQ score, not part of the study, scored in consensus [5]. The above DQ scale is similar to the four‐point (0‐to‐3) prostate imaging quality (PI‐QUAL) v2 scale used for assessment of T2SE image quality in which adequate/inadequate (1/0) scores are assigned to SNR, resolution, and lack of artifact and then summed [9]. This four‐point PI‐QUALv2 scale is now commonly used in evaluation of prostate T2SE image series [10, 11, 12]. We measured a Spearman coefficient of 0.83 in comparing the DQ and PI‐QUALv2 four‐point scales, indicating a very high correlation [6]. In both cases a DQ or PI‐QUAL value ≤ 1 generally warrants rescanning. The DQ evaluation in this work was done individually for each series, 29 each for the ADH and MOD sequences, 58 total. The signal ranges for each series were normalized to permit the same window and level for viewing. All identifying information was removed. The 58 series were then presented in random order to each reader.

The two image series (A, B) for each exam were also evaluated for preference using a five‐point scale:

−2 = strongly prefer Series A;
−1 = prefer Series A;
- 0 = prefer neither series;
- +1: prefer Series B;
- +2: strongly prefer Series B.

For each exam, the ADH and MOD series could be visualized side by side, but the order (A, B) was randomized and all descriptive annotation removed. For each exam, readers were also able to indicate any reason for preparing one series vs. the other.

### 2.3 Literature Review of T_2_ Values in Prostate

The average *T*_2_ values of healthy tissue and malignant lesions in the prostate at 3 T were determined from 12 representative prostate *T*_2_‐mapping studies [13, 14, 15, 16, 17, 18, 19, 20, 21, 22, 23] published in the MRI literature from 2009 to 2024, listed in Table S2. The average *T*_2_ value from each study was weighted by the number of participants to compute the literature‐average values from all studies. If a study reported a range or interval, instead of mean ± standard deviation (SD), the middle value of the range/interval was used.

### 2.4 Phantom Experiment

TE‐dependent effects were studied using the array of *T*_2_‐dependent spheres of the National Institute of Standards and Technology (NIST) standard MRI phantom [24]. An image of the slice containing the spheres is shown in Figure S1. The phantom was imaged using a conventional SE sequence and three different FSE sequences listed in Table 3. Two of these were the ADH and MOD sequences of Table 1 and the third used a longer echo spacing of 12.1 ms and smaller echo train length of 17. Each FSE sequence was acquired with TE_eff_ covering the range 10–200 ms in 10 ms steps, 20 scans in total. For conventional SE, only one phase encode was acquired per repetition at the desired TE value, using the same imaging parameters as the FSE acquisitions above where relevant. Analogous to FSE, the SE sequence was acquired with TE values of 10–200 ms in 10 ms steps. Throughout both the FSE and SE experiments, the receiver and transmit gains were kept fixed.

**TABLE 3:** Acquisition parameters of the Spin‐Echo (SE) and Fast‐Spin‐Echo (FSE) sequences for phantom study.

Parameter	FSE sequence 1	FSE sequence 2	FSE sequence 3	SE sequence
Freq. × phase directions^a^	S/I × L/R	S/I × L/R	S/I × L/R	S/I × L/R
Field‐of‐view (mm)	180 × 180	180 × 180	180 × 180	180 × 180
Slice thickness (mm)	3	3	3	3
In‐plane sampling (acquired)	320 × 280	320 × 280	320 × 280	320 × 280
In‐plane resolution (mm)	0.56 × 0.64	0.56 × 0.64	0.56 × 0.64	0.56 × 0.64
Repetition time (TR) (ms)	5000	5000	5000	5000
Refocusing flip angle (°)	130	130	130	—
TE (SE) or TE_eff_ (FSE) (ms)^b^	10–200, every 10	10–200, every 10	10–200, every 10	10–200, every 10
Acceleration factor	1	1	1	2
Number of averages	1	1	1	1
No phase wrap	Not used	Not used	Not used	Not used
Signal bandwidth (kHz)	±41.67	±27.78	±20.83	±15.26
Echo spacing (ESP) (ms)	8.3 (same as ADH)	10.2 (same as MOD)	12.1	—
Echo train length	24 (same as ADH)	21 (same as MOD)	17	—
Scan time (for each TE or TE_eff_)	1:05	1:15	1:35	11:40

^a^ S/I: superior/inferior; L/R: left/right.

^b^ TE_eff_ show prescribed values. The echo spacing and actual TE_eff_ are subject to the signal bandwidth.

For analysis, three vials with the values of *T*_2_ = 133.3, 96.9, and 64.1 ms (Vials #5, #6, and #7) were selected, as their *T*_2_ values (given by the specification [24]) were closest to those reported in the literature for normal and malignant prostate tissue (see Table S2). Specifically, signals between Vials #5 and #6 (*T*_2_ = 133.3 and 96.9 ms) were used to simulate the contrast between normal tissue and PCa for the peripheral zone (PZ), and signals for Vials #6 and #7 (*T*_2_ = 96.9 vs. 64.1 ms) for that between normal tissue and PCa in the transition zone (TZ).

### 2.5 T_2_ , T_2_ ,_eff_, and TE_opt_ Calculations for Phantom

For both the SE and FSE acquisitions, the mean signal magnitudes were measured as a function of TE (SE) or TE_eff_ (FSE) for each vial and fitted to an exponential decay to extract the apparent *T*_2_ values for each acquisition type. For each *T*_2_ sample, fitting the SE signals over TE yielded the estimated true *T*_2_ of the sample, and fitting the FSE signals over TE_eff_ yielded the effective *T*_2_ relaxation rate (*T*_2,eff_). For each pair of *T*_2_ vials being compared (#5 vs. #6; #6 vs. #7), the TE at which the SE contrast is expected to maximize (TE_opt_) was calculated using the measured *T*_2_ values via the following equation [25]:

(1) $${\mathrm{TE}}_{\mathrm{opt}}=\frac{T_{2,A}T_{2,B}}{T_{2,A}-T_{2,B}}\ln\left(\frac{\rho_{B}T_{2,A}}{\rho_{A}T_{2,B}}\right)$$

where $\rho_{A}=\rho_{B}=1$ was assumed for proton density. The TE_opt_ for FSE was computed in a similar way, with the $T_{2,\mathrm{eff}}$ values replacing *T*_2_ in Equation (1).

### 2.6 Data Collection for In Vivo Lesion Analysis

From July 11, 2024 to August 11, 2025, 48 subjects having a clinical prostate MRI exam were recruited to undergo an additional research MRI exam. An endorectal coil was not used. The clinical exam included diffusion weighted imaging (DWI) and dynamic contrast‐enhanced (DCE) MRI in addition to the T2W scans, all available for clinical interpretation by the radiologist. The resulting clinical report served as the basis for locating and segmenting lesions in the research exam. For each subject, the two exams were performed within 24 h of each other. The research exam did not use contrast material. If the clinical exam was performed first, then an overnight delay allowed contrast material clearance prior to the research exam. The clinical and research exams both included the MOD T2W sequence of Table 1 using TE_eff_ = 150. For this study, the research exam included additional runs of the MOD T2W sequence using TE_eff_ = 100 and additional values. Given that TE_eff_ = 100 ms and 150 ms were always acquired, the following additional TE_eff_ values were generally targeted: one around 125 ms (intermediate), one > 170 ms (high), and one < 100 ms (low), as allowed by the scan time constraint. The inter‐echo time and the exact TE_eff_ value of the FSE sequence were subject to scan plane angulation but always within ±5 ms of the prescription. For each subject, all MOD T2W scans were acquired with the same receiver gain, which was in fact the same for all subjects.

A subset of these 48 research exams was selected for lesion analysis. Inclusion criteria were: (i) the subject could not have previously undergone therapy of any kind for suspected prostate cancer, and (ii) the report for the clinical exam identified a specific, suspected malignant lesion in the prostate. Exams of all 24 subjects meeting the inclusion criteria were then included for lesion analysis. Demographics are shown in Table 2. All 24 subjects underwent biopsy, 18 subsequent to the research MRI exam, and the remaining six subjects having had a previous biopsy. From these 24 subjects, 31 lesions (21 in the PZ, 10 in the TZ) were identified for analysis. Additional details of the acquired TE_eff_ values, lesion count and locations, pathology findings, and segmentation areas are provided in Table S3.

### 2.7 In Vivo Lesion Segmentations

Referring to the clinical report produced by the clinical radiologist and the target lesion(s) specifically outlined in the clinical images, each suspected lesion for each subject was manually segmented by a radiologist (A.Ki.) using the TE_eff_ = 150 ms T2W image from the research exam. This segmentation served as the basis for segmenting the same lesion in all other TE_eff_ images of the same subject from that exam. Additionally, the surrounding healthy tissue within the same zone was also segmented in the same slice as the lesion. The mean signal magnitudes (S) were determined as a function of TE_eff_ for each segmented suspected PCa lesion ($S_{\mathrm{PCa}}$) and for its surrounding healthy tissue ($S_{\text{tissue}}$).

### 2.8 In Vivo Effective T_2_ (*T*_2,eff_) Analysis

The in vivo $S_{\mathrm{PCa}}$ and $S_{\text{tissue}}$ measurements as a function of TE_eff_ were fitted with an exponential curve to determine *T*_2,eff_ values of each segmented lesion and its surrounding healthy tissue. The distributions of the *T*_2,eff_ values in the segmented lesions and their surrounding healthy tissue were determined separately for PZ and TZ, along with the mean values. The mean *T*_2,eff_ values of the PCa lesions and the normal tissue were used to calculate the optimal TE_eff_ using Equation (1) as well as to plot the expected TE_eff_—dependent contrast behavior, separately for PZ and TZ.

### 2.9 In Vivo Lesion Contrast Analysis

The above $S_{\mathrm{PCa}}$ and $S_{\text{tissue}}$ measurements also yielded the following quantities for each lesion as a function of TE_eff_:

(2) $$\text{Contrast}=S_{\text{tissue}}-S_{\mathrm{PCa}},$$

(3) $$\text{Contrast}-\text{to}-\text{Noise Ratio}\;\left(\mathrm{CNR}\right)=\left(S_{\text{tissue}}-S_{\mathrm{PCa}}\right)/\sigma ,$$

(4) $$\text{Contrast}-\text{to}-\text{Tissue Ratio}=\left(S_{\text{tissue}}-S_{\mathrm{PCa}}\right)/S_{\text{tissue}},$$

where σ in Equation (3) denotes the image noise estimate determined using the algorithm described in the Supporting Information. For each of the metrics, the average across all lesions within the same zone was computed as a function of TE_eff_, after binning TE_eff_ values into 6 bins centered at 80, 100, 120, 150, 170, and 190 ms, each with a width of 20 ms. All metrics above were computed using raw signal values without any normalization. However, the CNR and the contrast‐to‐tissue ratio, which are normalized by the image noise and background tissue signal, respectively, serve as normalized contrast metrics that mitigate potential signal scaling differences across subjects.

### 2.10 Statistical Analysis

The difference scores in DQ and scores for preference were evaluated for significance for each individual reader and for the reader‐averaged scores using the Wilcoxon signed rank test [26] with significance taken as *p* < 0.05.

## 3 Results

### 3.1 Initial Reader Comparison at TE_eff_ = 150 ms Versus 100 ms

**FIGURE 2:**
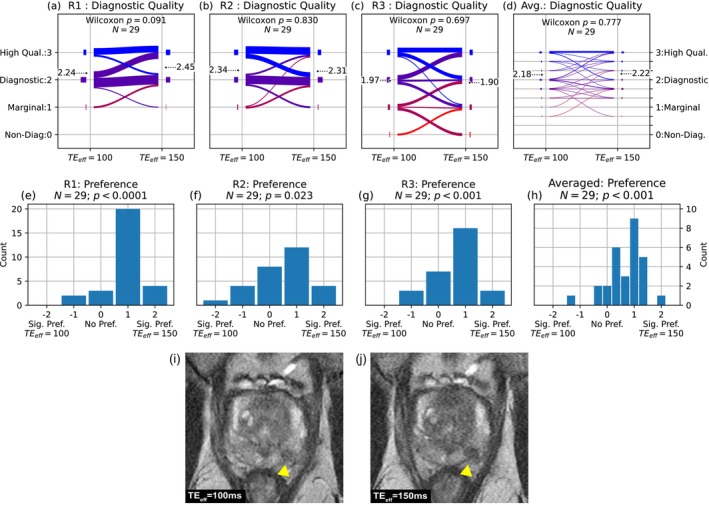
Reader study comparing T2W FSE prostate MRI using TE_eff_ = 100 versus 150 ms. (a–d) Diagnostic quality and (e–h) reader preference results are shown for individual readers (first three columns) and reader‐averaged (last column). Example depiction of a PCa lesion (arrow) from this evaluation are shown for (i) TE_eff_ = 100 ms and (j) TE_eff_ = 150 ms.

Figure 2 shows diagnostic quality scores and reader preference of individual readers and reader‐averaged. The distributions of the diagnostic quality scores are shown as histograms in Figure S2. There was no difference of statistical significance in diagnostic quality between the two sequences, but all three readers significantly preferred TE_eff_ = 150 ms over 100 ms. Readers R1, R2, and R3 commented on the improved perceived contrast of TE_eff_ = 150 ms in 27, 16, and 9 cases out of the 29 cases, with this improved contrast being the reason for its preference in 27, 15, and 7 cases, respectively. In the limited number of exams in which TE_eff_ = 100 ms was preferred, the reader comments generally ascribed this to reduced motion blurring. Figure 2i,j show example PCa depictions from this evaluation at TE_eff_ = 100 ms and 150 ms.

### 3.2 Literature Review of T_2_ Values in Prostate

Results of the literature survey are shown in Table S2. Literature‐average *T*_2_ values of normal tissue and malignant lesions are as follows: 150.3 ms and 87.0 ms in the peripheral zone, and 100.2 ms and 83.0 ms in the transition zone, respectively.

### 3.3 Phantom Experiments

**FIGURE 3:**
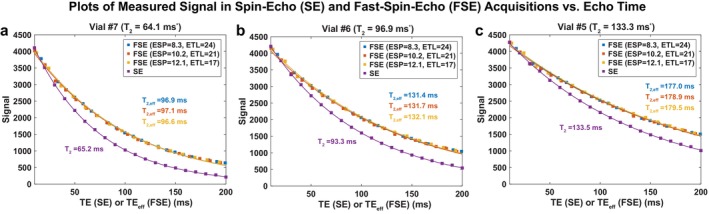
Plots of signal magnitude vs. TE (SE) or TE_eff_ (FSE) measured in vials of the *T*_2_ phantom using SE (orange) and FSE (blue) sequences. When the FSE sequence employs a constant flip angle as in clinical prostate T2W imaging, the signal undergoes an exponential decay characterized by an “effective” *T*_2_ (*T*_2,eff_) relaxation rate, which is longer than the “true” *T*_2_ relaxation rate observed with the SE sequence. Data are shown for (a) Vial #7 (*T*_2_ = 64.1 ms*), (b) Vial #6 (*T*_2_ = 96.9 ms*), and (c) Vial #5 (*T*_2_ = 133.3 ms*), chosen for their *T*_2_ values being closest to those previously reported for normal and malignant prostate tissue (seeTable S2). (*values according to the manufacturer specifications).

Figure 3 shows the SE and FSE signals from prostate‐like *T*_2_ samples as a function of TE (SE) and TE_eff_ (FSE)—specifically *T*_2_ array vials #5, #6, and #7 of the NIST phantom. For all vials, the fitted SE *T*_2_ values match the known *T*_2_ values within 0.002%–3.6%. More importantly, the plots demonstrate that the FSE signals can be closely approximated by an exponential decay at effective relaxation times *T*_2,eff_, that are on average 1.41× longer than the actual *T*_2_ values measured with the SE sequence. At the fixed refocusing flip angle (130°), the signal progressions were nearly identical for all three different FSE sequences despite having different echo train lengths and echo spacing.

**FIGURE 4:**
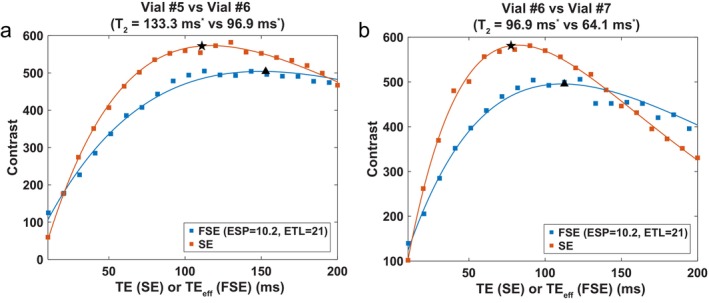
Plots of contrast vs. TE (SE) or TE_eff_ (FSE) from pairs of vials from the *T*_2_ phantom. (a) Vial #5 vs. Vial #6, simulating the peripheral zone and (b) Vial #6 vs. Vial #7, simulating the transition zone. The echo times of peak contrast in the SE and FSE scans were predicted from Equation (1) using the *T*_2_ and *T*_2,eff_ values of the vials, respectively, and are indicated by (★) and (▲). These closely match the echo times at which peak contrast was measured experimentally. (*values according to the manufacturer specifications).

Given their prostate‐like *T*_2_ values, these vials were paired to simulate lesion contrasts in the prostate PZ (Figure 4a) and TZ (Figure 4b), as a function of TE (SE) or TE_eff_ (FSE). The maximum SE contrast was observed at TE = 117.2 ms and 82.2 ms for the 133.3 versus 96.9 ms *T*_2_ pair (simulating PZ lesion) and 96.9 versus 64.1 ms *T*_2_ pair (simulating TZ lesion), respectively. Note that both values lie between the *T*_2_ values creating the contrast and agree within 5.6% of the TE_opt_ values (★ in Figure 4) computed using their *T*_2_ values in Equation (1). In contrast, the same pairs produced maximum FSE contrast at TE_eff_ = 150.0 ms and 111.2 ms, respectively, which lie outside the two *T*_2_ values creating each contrast. These TE_eff_ values that yielded the maximum FSE contrast, however, agreed within 1.8% with the predictions (▲ in Figure 4) from Equation (1) when the effective relaxation time values (*T*_2,eff_) shown in Figure 3 were used. In neither case did the optimal TE_eff_ lie between the two true *T*_2_ values of interest, as was the case for conventional SE.

### 3.4 In Vivo Effective T_2_ (*T*_2,eff_) Analysis

**FIGURE 5:**
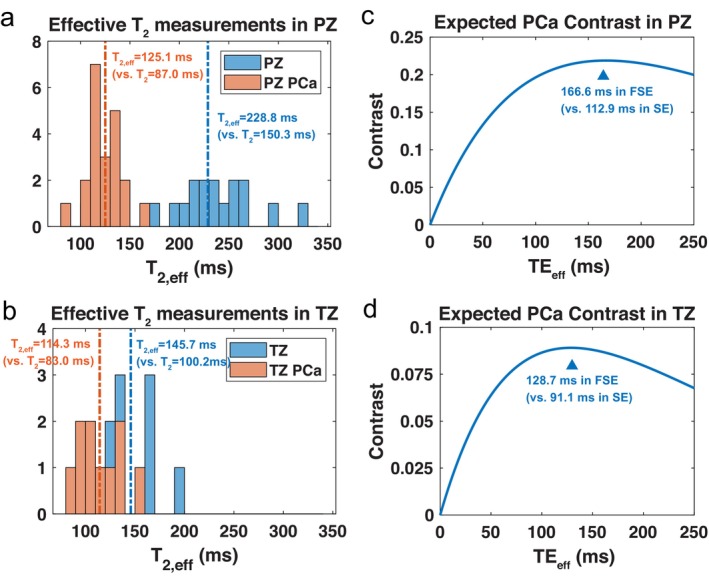
(a, b) Histogram of effective *T*_2_ times (*T*_2,eff_) for normal tissue (blue) and suspected PCa lesions (orange) measured in (a) peripheral zone (PZ) and (b) transition zone (TZ) using FSE acquisitions with different TE_eff_ values. In each case, the mean *T*_2,eff_ is shown, together with the mean conventional *T*_2_ (in parentheses) evaluated using the data of Table S2. (c, d) Plots of the expected contrast (signal difference) between normal tissue and PCa based on the mean *T*_2,eff_ values shown in (a, b), for (c) PZ and (d) TZ. In PZ, the contrast peaks at TE_eff_ = 166.6 ms per Equation (1) when using the mean *T*_2,eff_ values. This is markedly higher than the optimum TE of 112.9 ms predicted using the mean *T*_2_ values of 150.3 ms and 87.0 ms. Similarly, the contrast in TZ (d) is predicted to peak for the FSE acquisition at TE_eff_ = 128.8 ms.

The distributions of in vivo effective *T*_2_ (*T*_2,eff_) measurements from the segmented PCa lesions and the surrounding tissue are shown in Figure 5a,b. Compared to the literature‐average prostate *T*_2_ measurements (shown at the bottom of Table S2 and also in parentheses in Figure 5a,b), the mean $T_{2,\mathrm{eff}}$ measurements, or the “apparent” *T*_2_ relaxation rates observed in the FSE scans, are substantially longer, by a factor of 1.48 on average. These *T*_2,eff_ values were used to calculate the TE_eff_‐dependent signal progressions, whose differences yielded the expected contrast (signal difference) behaviors of the PCa lesion, shown as a function of TE_eff_ for the PZ and TZ in Figure 5c,d. The mean *T*_2,eff_ values of the healthy tissue and PCa lesions predict the optimal TE of the FSE scans to be 166.6 ms in the PZ and 128.7 ms in the TZ. These values are substantially higher than the corresponding 112.9 ms and 91.1 ms determined for the SE sequence using the literature *T*_2_ values. As in the phantom experiment, the optimal FSE TE_eff_ values lie outside the actual *T*_2_ values of interest, for the employed FSE sequence.

### 3.5 In Vivo Lesion Contrast Analysis

**FIGURE 6:**
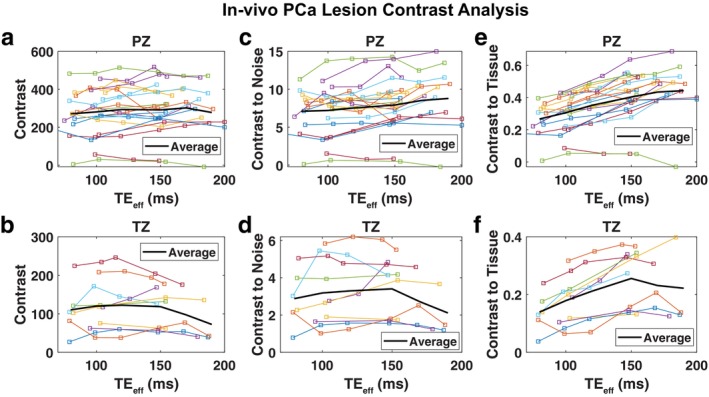
Plots of (a, b) lesion contrast, (c, d) lesion contrast‐to‐noise ratio (CNR), and (e, f) lesion contrast‐to‐background tissue ratio versus TE_eff_, for the 21 lesions identified in the peripheral zone (PZ) from 20 subjects (top row) and 10 lesions identified in the transition zone (TZ) from 10 subjects (bottom row). For all plots, the mean value is shown as the black line.

The in vivo analyses of the lesion contrast and the contrast‐to‐noise ratio (CNR) are shown in Figure 6a–d. In the PZ, both contrast and CNR show a modest increase from low TE_eff_ (e.g., 80 or 100 ms) to higher TE_eff_ values (e.g., 170 or 190 ms). In the TZ, however, the same metrics peak at TE_eff_ = 120 ms. Both results are consistent with the predicted contrast behavior of the PZ and TZ PCa lesions shown in Figure 5c,d.

The ratios of the contrast to background tissue are shown in Figure 6e,f. This metric within the PZ steadily increases with TE_eff_, primarily due to the diminishing tissue signal intensities at higher TE_eff_ values. In the TZ, however, this metric peaks at TE_eff_ = 150 ms despite a similar diminishing trend in the background tissue signal.

**FIGURE 7:**
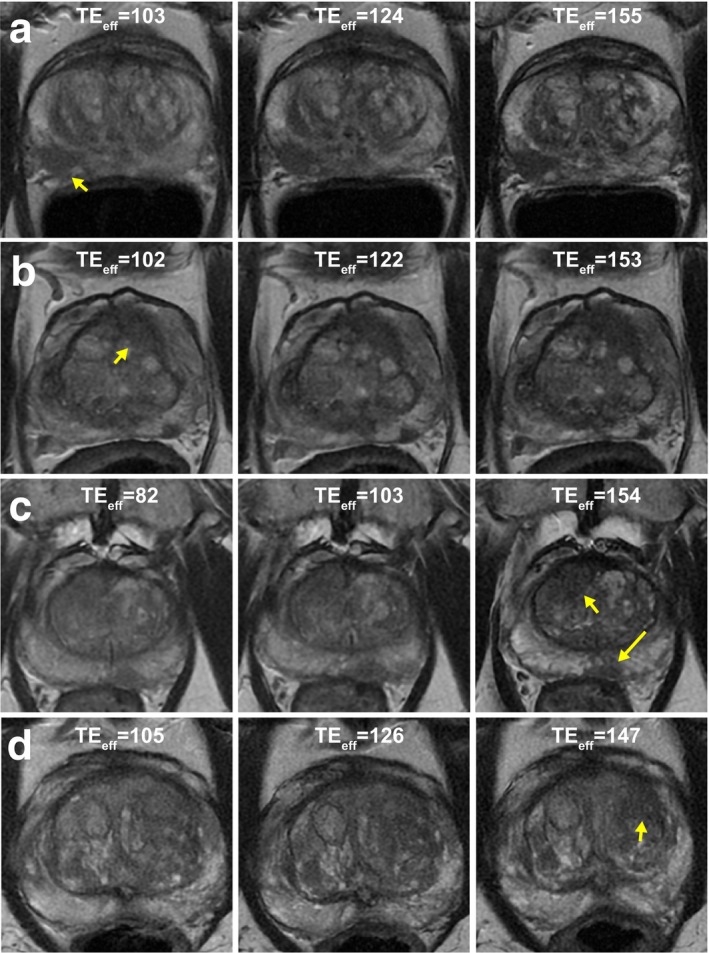
In vivo examples from four subjects with prostate cancer (PCa) imaged with different TE_eff_ values. For each subject shown, a biopsy was performed subsequent to the research MRI exam, and the Gleason Score (GS) is indicated. Lesions are identified with yellow arrows. (a) Peripheral zone (PZ) lesion, GS 4 + 3. (b) Transition zone (TZ) lesion, GS 3 + 4. (c) TZ lesion (short arrow), GS 3 + 4; PZ lesion (long arrow), GS 4 + 3. (d) TZ lesion, GS 3 + 3. In general, the visual contrast‐to‐background tissue ratio improves as TE_eff_ increases.

In vivo examples of lesion depiction at different TE_eff_ values are presented in Figure 7. In all cases shown in Figure 7, the analyzed lesion was biopsied subsequent to the research MRI exam.

## 4 Discussion

This study investigated the effect of TE_eff_ on PCa lesion depiction and contrast in T2W prostate imaging using an FSE sequence. The dependence of contrast on TE_eff_ has been largely unstudied, and the range of reported values is large across the published prostate studies.

The complicated echo pathways of FSE acquisition do not readily lend themselves to analysis, and it is tempting to use literature *T*_2_ values of interest in Equation (1) to calculate the optimal TE and set the TE_eff_ of the FSE sequence to this value. For SE imaging, assuming equal proton densities, the optimal TE lies between the *T*_2_ times of the two materials to be distinguished. However, with FSE acquisition, while the signal behavior of the vials also approximately follows an exponential decay, the relaxation time is extended to *T*_2,eff_. FSE contrast is again maximized at the TE_eff_ value predicted from Equation (1), but only when using *T*_2,eff_ values as inputs, yielding TE_eff_ values higher than and outside the range of the original *T*_2_ values in typical prostate T2W imaging.

It is worth noting that there also exist frameworks such as the Extended Phase Graph (EPG) algorithm [27, 28] capable of simulating signal evolutions and analytical modeling of echo‐time‐dependent signal/contrast behaviors in a FSE sequence. Our empirical in vivo measurements, however, provide practical clinical validation of the underlying physical principles.

Determination of the optimum TE_eff_ for the FSE acquisition depends on selection of the two target materials to be distinguished and their respective *T*_2,eff_ values. For prostate T2W MRI this simple determination becomes more complicated for multiple reasons. First, one wishes to simultaneously identify suspected malignancies in two zones, peripheral and transition, each with different relaxation times. Second, the reported values of *T*_2_ in normal and cancerous tissue in the peripheral and transition zones have broad ranges. These can make selection of an “optimum” TE_eff_ somewhat arbitrary. Nevertheless, both phantom experiments and in vivo measurements demonstrated that the *T*_2,eff_ relaxation times observed in the FSE acquisition were all substantially longer than their counterpart *T*_2_ values. This study suggests that this should be accounted for when choosing TE_eff_ for FSE‐based prostate T2W MRI.

The manner in which the observed signal decays with TE_eff_ in an FSE acquisition may depend on multiple FSE sequence parameters such as refocusing flip angle, echo train length, echo spacing, and phase encode ordering. It was beyond the scope of this study to investigate all possible variations of those parameters. However, the phantom experiment above confirms that the signal progression is largely independent of phase encode ordering (controlled by TE_eff_ prescription), echo train length, or echo spacing, indicating the generality of this study's results to other FSE sequences with the same flip refocusing angle. This study focused on a representative prostate T2W FSE sequence, which employed 21 echoes and a refocusing flip angle of 130°, the latter a value chosen to be small enough to provide adequately low Specific Absorption Rate (SAR). Higher refocusing angles would tend to reduce the optimal FSE TE_eff_ and bring it closer to the optimal SE TE, at the expense of increased SAR, while lower angles would extend optimal TE_eff_ to even higher values [29].

Partial volume effects can also provide some incentive for adopting higher TE_eff_ values. In practice, prostate T2W images are typically acquired with a 3 mm slice thickness and in‐plane resolution of 0.5 × 0.5 mm^2^, often making a PCa‐containing voxel a mixture of shorter‐*T*_2_ PCa lesion and longer‐*T*_2_ healthy tissue. Averaging over the comparatively large slice thickness makes the effective *T*_2_ of that voxel closer to that of the healthy tissue, pushing the optimal TE higher than that indicated by relaxation‐based calculation. This effect has been observed in a previous study which showed that the use of 1 mm slices provided improved lesion contrast versus 3 mm slices [30].

In this study, in addition to contrast and CNR, in vivo analysis also included contrast‐to‐tissue ratio. While CNR is a commonly used image quality metric in MRI, identification of PCa lesions involves detecting the contrast against the background healthy tissue. However, as opposed to the CNR, it is subject of further investigations to study how well the contrast‐to‐tissue ratio measures the ability to detect contrasting patterns from the local background, in the context of MRI and detection of PCa lesions.

Selection of the TE_eff_ in FSE involves assigning the multiple echoes acquired in the multi‐echo train to specific values along the phase encoding axis of k‐space. Changes to TE_eff_ are typically a simple modification, with negligible acquisition time penalty for the use of TE_eff_ near 150 ms versus lower values in an FSE acquisition composed of 20 or more echoes per excitation. For example, the 2 min 18 s scan time of the MOD sequence remained unchanged when its TE_eff_ was varied over 10–200 ms in this study.

### 4.1 Limitations

This work has several limitations. First, the sequence parameters used in the initial comparison of TE_eff_ = 150 ms versus 100 ms were not identical. However, the two sequences did have equivalent in‐plane resolution and SNR, and in the vast majority of cases resulted in interpretable images. Moreover, the phantom studies showed equivalent behavior of the two sequences vs. TE_eff_. Thus, the preference for TE_eff_ = 150 ms can be largely attributed to the increased perceived contrast at TE_eff_ = 150 ms. We have subsequently adjusted the MOD sequence to have TE_eff_ = 150 ms, and when compared to the ADH sequence (also with TE_eff_ = 150 ms), shown it to be superior because of reduced sensitivity to motion [6].

Second, not all analyzed lesions were biopsied after the research exam. However, those not biopsied after the research exam had been biopsied previously. Third, the in vivo lesion contrast measurements at multiple TE_eff_ values are subject to non‐identical slices due to subtle inter‐series motion, providing uncertainty in the analysis. The in vivo lesion analysis was limited in the number of lesions studied, with no subdivision according to PI‐RADS score. The lesion segmentation was performed by only one radiologist (according to the corresponding clinical report).

## 5 Conclusion

In this study of prostate T2W FSE MRI, TE_eff_ = 150 ms was preferred over 100 ms. This is due to the apparent *T*_2_ in the FSE sequence being longer than the true *T*_2_, resulting in the effective echo time (TE_eff_) for maximum contrast being longer than that calculated using exponential modeling of a simple spin‐echo sequence with literature *T*_2_ values.

## Funding

This work was supported by National Institutes of Health, (C06 RR018898, R01 EB031790).

## Ethics Statement

Research MRI exams were performed under Protocol 15‐003511 approved by the Institutional Review Board under which all participants gave their written informed consent.

## Consent

All participants gave their consent for their clinical information to be made available for research purposes.

## Conflicts of Interest

The authors declare no conflicts of interest.

## Supporting information


**Table S1:** List of 45 published prostate MRI studies from 2004 to 2024 included in the survey of the TE_eff_ values for T2‐weighted fast (or turbo) spin echo sequences shown in Figure 1.
**Table S2:** Prostate T2 relaxation rate values reported in literature for PZ, PZ PCa, TZ, and TZ PCa. All results are for 3 T. (PZ: peripheral zone, TZ: transition zone, PCa: prostate cancer, + Confidence Interval, * Range, ^ Inter‐quartile Range, ** Average weighted by the number of participants).
**Table S3:** List of acquired TEeff values, lesion locations, sizes of the lesion and tissue segmentations, and biopsy results in the in vivo lesion analysis. The segmented areas are given as the mean values across the acquired TEeff values. Each pixel had an area of 0.098 mm2, making roughly 10 pixels correspond to an area of 1 mm2. Biopsy results are shown as Pre‐ or Post‐ the research MRI exam with the companion Gleason score (GL) or benign.
**Figure S1:** Image of the phantom containing the vials with known T2 relaxation times, acquired using the FSE sequence of Table 2. Each vial is individually identified with its vial number (black font) and its actual T2 values (blue font) as calibrated by the vendor.
**Figure S2:** Histograms of the Diagnostic Quality (DQ) scores, shown for each reviewer (R1, R2, and R3) and reader‐averaged. (WSR: Wilcoxon Signed Rank, μ: mean value).
