# Supplementary material for: Optimal Selection of the Effective Echo Time (TEeff ) for T2‐Weighted Fast‐Spin‐Echo MRI of the Prostate at 3.0 T: Effect on Lesion Conspicuity
Source: J Magn Reson Imaging. 2026 Apr 23;64(3):616–28. doi: 10.1002/jmri.70351 (PMC13466591; doi:10.1002/jmri.70351)
Supplement: Supplementary file 1 [file JMRI-64-616-s001.docx]

**SUPPLEMENTAL MATERIALS**

| **Year** | **Authors** | **Journal** | **Citation** | **Field Strength (T)** | **TE_eff_ (ms)** |
| --- | --- | --- | --- | --- | --- |
| 2004 | Mullerad et al. | Radiology | 232:140-146 | 1.5 | 96 |
| 2006 | Akin et al. | Radiology | 239:784-792 | 1.5 | 96-120 |
| 2007 | Miao et al. | Eur J Radiol | 61:297-302 | 3.0 | 96 |
| 2010 | Villeirs et al. | Eur J Radiol | 77:340-345 | 1.5 | 139 |
| 2011 | Tamada et al. | Am J Roentgenol | 197:664-670 | 1.5 | 102 |
| 2011 | Roethke et al. | Eur J Radiol | 79:189-195 | 1.5 | 121 |
| 2012 | Rosenkrantz et al. | Am J Roentgenol | 199:830-837 | 3.0 | 123 |
| 2012 | Turkbey et al. | BJUI | 110:1642-1647 | 3.0 | 120 |
| 2012 | Valentini et al. | Eur J Radiol | 81:3090-3095 | 1.5 | 85 |
| 2012 | Ruprecht et al. | Eur J Radiol | 81:456-460 | 1.5 | 109 |
| 2013 | Tempany et al. | JMRI | 37:1035-1054 | 3.0 | 102 |
| 2013 | Rosenkrantz et al. | JMRI | 38:694-700 | 3.0 | 123 |
| 2013 | Bomers et al. | Radiology | 268:451-460 | 1.5 | 104 |
| 2014 | Kim et al. | Radiology | 271:435-444 | 1.5 | 104 |
| 2014 | Wang et al. | JMRI | 39:781-788 | 3.0 | 115 |
| 2015 | Rosenkrantz et al. | JMRI | 43:990-997 | 3.0 | 105 |
| 2015 | Jambor et al. | JMRI | 41:1394-1404 | 3.0 | 101 |
| 2016 | Stanzione et al. | Eur J Radiol | 85:2269-2274 | 3.0 | 101 |
| 2017 | Weiss et al. | JMRI | 46:1507-1515 | 3.0 | 92 |
| 2017 | Benndorf et al. | Eur J Radiol | 93:9-15 | 1.5 | 110 |
| 2017 | Becker et al. | Eur J Radiol | 94:58-63 | 3.0 | 93 |
| 2018 | Smith et al. | JMRI | 49:1694-1703 | 3.0 | 120 |
| 2018 | Nayak et al. | JMRI | 47:176-185 | 3.0 | 105-125 |
| 2018 | Benndorf et al. | Eur J Radiol | 99:62-67 | 1.5 | 110 |
| 2018 | Campli et al. | Eur J Radiol | 101:17-23 | 1.5 | 120 |
| 2019 | Tong et al. | JMRI | 50:1614-1619 | 3.0 | 90 |
| 2019 | Cuocolo et al. | Eur J Radiol | 116:144-149 | 3.0 | 101 |
| 2019 | Schaudinn et al. | Eur J Radiol | 116:180-185 | 3.0 | 126 |
| 2020 | Cui et al. | JMRI | 52:552-564 | 3.0 | 88 |
| 2020 | Tamada et al. | JMRI | 53:283-291 | 3.0 | 95 |
| 2020 | Wei et al. | Eur J Radiol | 127:108977 | 3.0 | 100 |
| 2020 | Krauss et al. | Eur J Radiol | 129:109063 | 3.0 | 104 |
| 2021 | Youn et al. | Eur J Radiol | 142:109894 | 3.0 | 80-100 |
| 2021 | Tamada et al. | Eur J Radiol | 121:108704 | 3.0 | 95 |
| 2021 | Gassenmeier et al. | Eur J Radiol | 137:109600 | 3.0 | 104 |
| 2021 | Kim et al. | Eur J Radiol | 145:110012 | 3.0 | 103 |
| 2021 | Wang et al. | Abdl Radiol | 46:3378-3386 | 3.0 | 133-150 |
| 2022 | Park et al. | JMRI | 55:1735-1744 | 3.0 | 95 |
| 2022 | Johnson et al. | JMRI | 56:184-195 | 3.0 | 100 |
| 2022 | Jung et al. | BJR | 95:20211378 | 3.0 | 103 |
| 2023 | Bischoff et al. | Radiology | 308:00:00 | 3.0 | 120 - 133 |
| 2023 | Kelleher et al. | JMRI | 58:620-629 | 3.0 | 89 |
| 2023 | Tong et al. | JMRI | 58:1055-1064 | 3.0 | 100 |
| 2023 | Lee et al. | Eur J Radiol | 166:111017 | 3.0 | 97-107 |
| 2024 | Oerther et al. | Eur Radiol | 34:7053-7062 | 3.0 | 104 |

* For papers with a range of TE values, the middle value of the range is used for the histogram in Fig. 1a.

Supplemental Table S1. List of 45 published prostate MRI studies from 2004 to 2024 included in the survey of the TE_eff_ values for T2-weighted fast (or turbo) spin echo sequences shown in Fig. 1.

| **Authors** | **Year** | **No. of Participants** | **PZ (ms)** | **PZ PCa (ms)** | **TZ (ms)** | **TZ PCa (ms)** |
| --- | --- | --- | --- | --- | --- | --- |
| Gibbs et al. [13] | 2009 | 20 | 142 ± 24 | n/a | 109 ± 20 | n/a |
| Yamauchi et al. [14] | 2015 | 36 | 169.6 ± 38.7 | 85.4 ± 12.3 | n/a | n/a |
| Dinh et al. [15] | 2015 | 16 | 136 ± 31 | 106 ± 26 | 95 ± 17 | 86 ± 8 |
| Dinh et al. [15] | 2015 | 67 | 139 ± 26 | 107 ± 18 | 96 ± 13 | 85 ± 16 |
| Dregely et al. [16] | 2016 | 17 | 129 ± 39 | 72 ± 14 | 83 ± 12 | 72 ± 14 |
| Giganti et al. [17] | 2017 | 20 | 133.7 (99.6-152.4)^^^ | 83.5 (70.1-95.8)^^^ | 97.7 (84.4-106)^^^ | 83.5 (70.1-95.8)^^^ |
| Sabouri et al. [18] | 2017 | 18 | 138 (68-298)^*^ | 94 (46-187)^*^ | 138 (65-276)^*^ | 101 (39-158)^*^ |
| Sabouri et al. [19] | 2017 | 17 | 114 (58-295)^*^ | 84 (30-139)^*^ | 107 (57-237)^*^ | 89 (41-120)^*^ |
| Van Houdt et al. [20] | 2018 | 12 | 149 (111-223)^*^ | 78 (67-105)^*^ | 91 (70-109)^*^ | 78 (67-105)^*^ |
| Hepp et al. [21] | 2022 | 55 | 193 ± 58 | 80 ± 13 | n/a | n/a |
| Klingebiel et al. [22] | 2022 | 44 | 141 (119-171)^+^ | 84.6 (79.1-96.2)^+^ | 96.8 (90.9-113)^+^ | 74.8 (70.4-77.7)^+^ |
| Bucher et al. [23] | 2024 | 54 | 151.7 (146.9-156.5)^*^ | 73.5 (67.9-79.1)^*^ | n/a | n/a |
| Average** |  |  | 150.3 | 87.0 | 100.2 | 83.0 |

Supplemental Table S2. Prostate *T*_2_ relaxation rate values reported in literature for PZ, PZ PCa, TZ, and TZ PCa. All results are for 3 T. (PZ: peripheral zone, TZ: transition zone, PCa: prostate cancer, ^+^ Confidence Interval, ^*^ Range, ^^^ Inter-quartile Range, ^**^ Average weighted by the number of participants)

| **Subject No.** | **TE_eff_ acquired (ms)** | **Lesion Location(s),**  **Lesion / Tissue Segmentation Areas (mm^2^)** | **Pathology** |
| --- | --- | --- | --- |
| 1 | 103,124,145,146 | PZ (60 / 485) | Post; GL 4+3 |
| 2 | 106,117,138,159 | PZ (279 / 90) | Pre; GL 3+4 |
| 3 | 102,122,143,153 | TZ (98 / 494) | Post; GL 3+4 |
| 4 | 103,124,145,155 | PZ (26 / 242) | Post; GL 3+3 |
| 5 | 103,154 | TZ (57 / 373) | Post; GL 4+3 |
| 6 | 103,154 | PZ (24 / 287) | Post; GL 3+4 |
| 7 | 105,126,147 | PZ (67 / 369)  TZ (126 / 1129) | Post; GL 3+3 Post; GL 3+3 |
| 8 | 99,129,149 | PZ (12 / 190) | Pre; GL 3+4 |
| 9 | 82,103,154 | PZ (42 / 272)  TZ (64 / 496) | Post; GL4+3 Post; GL 3+4 |
| 10 | 84,95,116,148 | PZ (29 / 276) | Pre; GL 3+4 |
| 11 | 79,98,118,147 | TZ (156 / 331) | Post; GL3+3 |
| 12 | 83,104,115,146,167 | TZ (106 / 1209)  PZ (26 / 373) | Post; GL3+4 Post; GL 3+4 |
| 13 | 75,97,118,150,172 | PZ (76 / 433) | Post; GL 3+4 |
| 14 | 79,99,118,148,168,187 | PZ (20 / 711)  TZ (41 / 379)  PZ (27 / 563) | Pre; GL3+3 Pre; GL 3+4 Pre; GL3+3 |
| 15 | 79,99,119,149,169,188 | PZ (17 / 239)  TZ (35 / 184) | Post; benign Post; benign |
| 16 | 83,104,125,146,177 | PZ (39 / 283) | Pre; GL 3+4 |
| 17 | 85,117,148,170,191 | PZ (51 / 819) | Post; GL 3+3 |
| 18 | 81,101,121,151,182 | PZ (45 / 259) | Post; GL 3+3 |
| 19 | 96,128,149,181 | PZ (23 / 382) | Pre; GL 3+4 |
| 20 | 82,102,154,184 | TZ (231 / 359)  PZ (61 / 188) | Post; GL 3+4 Post; GL 3+4 |
| 21 | 95,148,179 | TZ (29 / 484) | Post; benign |
| 22 | 100,130,150,180 | PZ (14 / 855) | Post; GL 3+4 |
| 23 | 102,153,204 | PZ (26 / 260) | Post; benign |
| 24 | 53,96,149,203 | PZ (32 / 201) | Post; GL 3+3 |

Supplemental Table S3. List of acquired TE_eff_ values, lesion locations, sizes of the lesion and tissue segmentations, and biopsy results in the in-vivo lesion analysis. The segmented areas are given as the mean values across the acquired TE_eff_ values. Each pixel had an area of 0.098 mm^2^, making roughly 10 pixels correspond to an area of 1 mm^2^. Biopsy results are shown as Pre- or Post- the research MRI exam with the companion Gleason score (GL) or benign.

Supplemental Figure S1. Image of the phantom containing the vials with known *T*_2_ relaxation times, acquired using the FSE sequence of Table 1. Each vial is individually identified with its vial number (black font) and its actual *T*_2_ values (blue font) as calibrated by the vendor.

Supplemental Figure S2. Histograms of the Diagnostic Quality (DQ) scores, shown for each reader (R1, R2, and R3) and reader-average. (WSR: Wilcoxon Signed Rank, *μ*: mean value)

*Image Noise Estimation for In-vivo Lesion Analysis*

Image noise was estimated from each T2W image as the standard deviation (S.D.) within a circular ROI that placed in the most homogeneous area of the T2W image set. The developed algorithm exploits the idea that the ROI location that minimizes magnitude S.D. within the ROI gives the best estimate of the image noise, given that any signal variations from tissue will only increase the S.D. value. Let *n* denote the number of TEs at which respective images are acquired, and $S_{i}(x,y)$ denote the signal magnitude for the *i*^th^ TE, where $i=1,\cdots, n$. First, the algorithm prepares a S.D. map $\sigma_{i}(x,y)$ whose value is the S.D. of $S_{i}(x,y)$ within the circular ROI centered at $(x,y)$. For a single TE image, the smallest value in this S.D. map identifies the most homogeneous area in that image. Given the multiple TE images to examine in this study, the algorithm instead identified the most homogeneous location across all TE images by finding the location $\left( \hat{x},\hat{y} \right)$ that minimizes the value of the S.D. map summed over all TEs: $\sigma(x,y)=\sum_{i=1}^{n} \sigma_{i}(x,y)$. This approach not only eliminates human errors in the manual ROI placements but also systematically ensures that the chosen ROI location has fewest contributions from tissue signal variations. Once $\left( \hat{x},\hat{y} \right)$ is determined, the noise values for each of the *n* TE images were given by $\sigma_{i}(\hat{x},\hat{y})$ for $i=1,\cdots, n$.

Coil sensitivity correction makes the noise level non-uniform in the reconstructed images. Thus, the search for $\left( \hat{x},\hat{y} \right)$ was limited to the center 80x50cm of the image around the prostate, which consistently resulted in ROI locations within the obturator internus muscle as shown below. The ROIs were a circle of 30-voxel (9.5mm) diameter with 697 voxels, for sufficient noise sampling.
